# Determining the potential use of biosurfactants in preventing endodontic infections

**DOI:** 10.1111/eos.12900

**Published:** 2022-11-03

**Authors:** Zahraa Amer Hashim, Jean‐Yves Maillard, Melanie Jay Wilson, Rachel Jane Waddington

**Affiliations:** ^1^ Department of Clinical Laboratory Science, College of Pharmacy Mosul University Nineveh Iraq; ^2^ Cardiff School of Pharmacy and Pharmaceutical Sciences Cardiff University Cardiff Wales UK; ^3^ School of Dentistry Cardiff University Cardiff Wales UK

**Keywords:** anti‐adhesive, anti‐microbial, endodontic, Lactobacillus plantarum, rhamnolipid

## Abstract

Microbial biofilms play a dominant role in the failure of endodontic therapies. Bacterial adhesion is the first step in the establishment of biofilms, activating the host immune response leading to tissue damage. Biosurfactants are microbe‐derived tensioactive molecules with latent anti‐adhesive and anti‐microbial activity. This study reports the extraction and characterization of a biosurfactant from *Lactobacillus (L.) plantarum* (*Lp*‐BS) and investigates its anti‐microbial and anti‐adhesive properties compared to rhamnolipid, a commercially available biosurfactant. *Lp*‐BS, extracted from *L. plantarum* during the growth phase, was characterized as a glycoprotein, able to reduce surface tension and emulsify non‐polar liquids. Proteomic analysis of *Lp*‐BS identified three bacterial adhesin‐like proteins, suggesting roles in hindering bacterial adhesion. *Lp*‐BS did not show significant anti‐microbial activity against endodontic pathogens from the *Streptococcus (Strep.) anginosus* group or *Enterococcus (Ent.) faecalis* at 50 mg/ml. However, anti‐adhesive activity on abiotic surfaces was observed against both *Strep. anginosus* and *Strep. intermedius*. Rhamnolipid exhibited strong anti‐microbial activity, with minimum inhibitory concentrations of 0.097 mg/ml against *Strep. anginosus*, and 0.048 mg/ml against *Strep. constellatus* and *Strep. intermedius*, in addition to a marked anti‐adhesive activity. These findings offer preliminary evidence for the potential application of biosurfactants as an anti‐microbial and/or anti‐adhesive pharmacotherapy in endodontics.

## INTRODUCTION

Pulp tissue that is subjected to partial destruction due to deep caries, trauma, or failed restorative measures, can be preserved by means of vital pulp therapy. Such therapy depends on the regenerative ability of the dentine‐pulp complex, in promoting tissue healing and inducing reparative dentine formation. If pulp therapy fails, non‐vital teeth may be managed by the placement of inert filling materials within root canals. The success of both vital pulp therapy and root canal treatment is dependent on ensuring low levels of microbial contamination [1]. Despite the diverse microbial community involved in dental infections, members of the commensal *Streptococcus (Strep.) anginosus* group have been documented as pioneer colonisers implicated in the initiation of pulpitis [2]. *Enterococcus (Ent.) faecalis* has also been recognised as a causative agent in persistent endodontic infections and pulpitis [3].

Currently, the most commonly used canal irrigant is sodium hypochlorite, a potent disinfectant with documented prophylactic protection against microbial colonisation of the pulp and root canals [1]. However, inappropriate use of sodium hypochlorite can result in severe painful tissue reactions appearing as swelling and haemorrhage and can progress into secondary infection [4]. Moreover, a substantial validation for its use in vital pulp therapy has been recommended [5]. It has been documented that a combination of more than one irrigant with surface active agents is required for more efficient debridement and decontamination of the canals. The enhanced microbial killing potential obtained by the addition of biosurfactants has attracted much attention as potential broad‐spectrum non‐toxic anti‐microbials [6, 7]. The inherent properties of biosurfactants synthesised and released by probiotic bacteria, such as those produced by the genus *Lactobacillus (L.)*, possibly related to the flagging of the cohesive forces between bacterial cell membranes and extracellular polymeric substances [8] and these unique structure‐function characteristics of biosurfactants, have provided many opportunities to explore their industrial and biomedical applications [9, 10]. Their potential as effective anti‐microbials for endodontic treatment remains, however, unexplored.

Chemically, biosurfactants are diverse amphiphilic compounds with hydrophilic and hydrophobic moieties that have the ability to affect surface properties by reducing surface tension and emulsifying immiscible phases [11, 12, 13]. These compounds have been grouped according to their molecular weight into low molecular weight biosurfactants consisting of glycolipids and lipopeptides and high molecular weight polymeric biosurfactants [14]. The anti‐microbial activity of microbial‐derived biosurfactants has been reported, including that of rhamnolipids from *Pseudomonas aeruginosa* [15], surfactin and iturin produced by *Bacillus subtilis* strains [16], mannosylerythritol lipids from *Candida antarctica* [17], and biosurfactants isolated from *Strep. thermophilus* A and *Lactococcus lactis 53* [18, 19].

The ability of lactobacilli to produce biosurfactants has been widely reported, although very little information is available regarding *L*. biosurfactants’ chemical characteristics which might impact their production for commercial purposes. *L*. biosurfactants have been partially characterised as multipotent complexes of proteins, polysaccharides and/or lipids [20, 21, 22]. Based on their documented anti‐microbial and surface alteration properties, microbial‐derived biosurfactants may have potential application for endodontic treatment. In this context, the aim of this study was first to investigate *L. plantarum* biosurfactant production, extraction, and chemical characterisation, and second to determine the anti‐microbial and anti‐adhesive properties of the extracted biosurfactants against selected common endodontic bacterial pathogens.

## MATERIAL AND METHODS

### Probiotic and pathogenic strains

*L. plantarum* NCIMB8826 was grown in 5% v/v CO_2_ at 37°C on de Man, Rogosa and Sharpe (MRS) agar or in broth. Three *Streptococcus* spp. of the anginosus group (*Strep. anginosus* 670/95, *Strep. constellatus* S08‐07, and *Strep. intermedius* HW13) and *Ent. faecalis* RB17 (representing endodontic pathogens) were cultured on fastidious anaerobe agar (FAA) (Lab M) supplemented with 5% (v/v) defibrinated horse blood in 5% v/v CO_2_ at 37°C.

*Staphylococcus (Staph.) aureus* NCTC8325 and *Escherichia (Esh.) coli* NCTC10418 (representing commonly Gram‐positive and Gram‐negative test bacteria) were cultured on FAA agar (Lab M) supplemented with 5% (v/v) defibrinated horse blood at 37°C. Bacterial suspensions were prepared by inoculation of colonies from FAA plates in brain–heart infusion (BHI) broth (Oxoid). Broth cultures of the probiotic and pathogenic strains were grown overnight at 37°C in 5% CO_2_ before being pelleted, washed, and re‐suspended in phosphate‐buffered saline (PBS). The absorbance readings were adjusted using a spectrophotometer to 0.08–0.1 at 600 nm corresponding to 1–1.5 × 10^8^ CFU/ml. Rhamnolipid (biological origin *P. aeruginosa*) was obtained pre‐prepared from a commercial source (Sigma‐Aldrich).

### Growth of *L. plantarum* for biosurfactant production and extraction

Studies have described how biosurfactants synthesised by microorganisms may either be released into the growth medium or remain attached to the bacterial cell wall [14]. An overnight preculture of *L. plantarum* in MRS broth was diluted in 400 ml broth to a final concentration of around 1 × 10^7^ CFU/ml and further incubated at 37°C in 5% CO_2_ for up to 48 h. In order to determine the optimal time for biosurfactant production, a standard growth curve was generated, and four harvest time points were chosen: 8.5 h post incubation (representing the mid‐exponential phase), 16 h (representing the early stationary phase), and 24 h and 48 h (representing the late stationary phase). Harvested samples were ultracentrifuged at 10,000 *g* at 10°C for 10 min. The supernatant containing biosurfactant was decanted, passed through 0.22 μm filter unit (Whatman) and tested for surface tension measurements. The bacterial pellet was washed twice with 400 ml sterile double distilled water, resuspended in PBS (PBS: 10 mM KH_2_PO_4_/K_2_HPO_4_, pH adjusted to 7.0) and gently stirred at room temperature (20–25°C) for 18 h. Cell‐bound biosurfactant released into the PBS (termed hence forth as *Lp*‐BS) was recovered by centrifugation (10,000 *g*, 10°C for 10 min) with the resultant supernatant filtered through a 0.22 μm filter unit [23]. *Lp*‐BS was lyophilised to concentrate the biosurfactant and then dialysed against double distilled water (using dialysis membrane with molecular weight cut‐off 1000 Da) at 4°C for 48 h. The dialysate was filter sterilised, lyophilised, and weighed to calculate biomass yield, and stored at −20°C.

### Surface tension activity determination

Biosurfactants are typically ascribed characteristics, which involve abilities to lower the surface and interfacial tension between different phases (liquid–air, liquid–liquid, and liquid–solid). Hence, as an indicator of biosurfactant production, the surface tension of the bacterial supernatant and the *Lp*‐BS cell‐bound PBS‐extract was measured at room temperature using the du Nouy ring method, incorporating a 1.9 cm du Nouy platinum ring into a dynamic contact angle analyser [17]. Uninoculated MRS broth provided background reference value for bacterial supernatant while PBS was used for the cell‐bound PBS extract. All analyses were performed in triplicate.

### Emulsifying activity determination

Emulsification index of *Lp*‐BS was determined against six hydrocarbons (kerosene, heptane, hexane, xylene, motor oil, and sunflower oil). Two millilitres of *Lp*‐BS solution (dissolved 1 mg/ml in distilled water) was added to 2 ml of one of the six hydrocarbons in glass test tubes, which were then vortexed at high speed for 2 min and left on the bench for 24 h at 25°C. Tween 80 (representative of a non‐ionic surfactant and emulsifier) and distilled water were used as positive and negative controls, respectively. The emulsification indexes (EI24) were calculated as the percentage of the height of the emulsified layer (mm) divided by the total height of the liquid column (mm) [24]. All analyses were performed in triplicate.

### Characterisation of *Lp*‐BS using Fourier transform infrared (FT‐IR) spectroscopy

The presence of specific functional groups within the *Lp*‐BS was examined using a Nicolet 380 Fourier transform infrared (FT‐IR) T‐IR‐4100 Spectrometer. Samples were prepared by homogenous dispersion of 30 mg of *Lp*‐BS in potassium bromide (KBr) powder (ratio of 1:100) and formation of a translucent pellet using a hand‐press providing 7500 kg of pressure. Spectra were obtained from 64 scans with a resolution of 8 cm in the range of 600−4000 cm^−1^. A KBr pellet provided a background reference that was subtracted from the IR spectra [25]. This assay was repeated thrice.

### Lipid detection by thin layer chromatography

To assess for lipid moieties in the *Lp*‐BS preparation, 100 μl of *Lp*‐BS solution (1 mg/ml in distilled water) was spotted 1.5 cm from one edge of a precoated silica gel plate (5 cm × 10 cm, Sigma‐Aldrich). Rhamnolipid (1 mg/ml) (Sigma‐Aldrich) was also spotted onto the plate as a positive control. The edge of the plate was placed vertically in a developing jar and dipped to touch a solvent mixture (mobile phase) of chloroform (Fisher Scientific), methanol (Fisher Scientific), and acetic acid (Fisher Scientific) (65:15:2 v/v), which was allowed to be drawn up through the silica plate via capillary action. When the mobile phase approached near the top end of the plate, the separated components were detected by spraying the plate with 0.42% w/v ammonium molybdate and 0.2% w/v cerium (IV) sulphate in 6.2% v/v sulphuric acid which was then heated in the oven (120°C for 10 min) [13]. The assay was performed in triplicate.

### Bicinchoninic acid protein assay (BCA)

Total protein content in the *Lp*‐BS was quantified using a Pierce BCA Protein Assay Kit (Thermo Fisher Scientific) following manufacturer's instructions and measured against standard curves created using bovine serum albumin (Sigma). Analyses were performed in triplicate.

### Carbohydrate determination using the anthrone assay

Crystalloid anthrone (0.2 g) was dissolved in 71 ml concentrated >99.7% sulphuric acid in an ice bath and then made up to 1 L with deionised distilled water. Standard samples were prepared using glucose (0–200 μg/ml) in double distilled water, and 0.5 ml of *Lp*‐BS (1 mg/ml) or standard was mixed well with 2.5 ml of anthrone reagent in a stoppered glass tube and heated to 95°C in a water bath for 10 min. Absorbance at 620 nm was measured and total carbohydrate concentration was calculated from calibration curves created from readings of the glucose standards [26]. Data were expressed as mean ± standard deviation (SD) of three independent repeats.

### Mass spectrometric analysis of *Lp*‐BS

Mass spectrometric analysis was performed at the Proteomics Facility, University of Bristol. Fifty micrograms of *Lp*‐BS was electrophoretically separated by sodium dodecylsulfate‐polyacrylamide gel electrophoresis (SDS‐PAGE) and the resulted gel was stained with colloidal Coomassie blue stain. Ten bands showing intense staining were excised from the gel (noting their migratory position) and placed in individual microcentrifuge tubes. In‐gel tryptic digestion was performed on each gel slice and the resultant peptides were dried by evaporation, re‐suspended in 1% formic acid and fractionated using an Ultimate 3000 nano‐LC system in line with an LTQ‐Orbitrap Velos mass spectrometer (Thermo Scientific). Proteome Discoverer software v1.4 (Thermo Scientific) was used to process and quantify the obtained raw data files. Searches for protein identification were made against the UniProt *L. plantarum* database (28,578 sequences) using the SEQUEST algorithm where the peptide precursor mass tolerance was set at 10 ppm, and MS/MS tolerance was set at 0.8 Da. Search standards comprised carbamidomethylation of cysteine (+57.0214) as a fixed modification and oxidation of methionine (+15.9949) as a variable modification. Full tryptic digestion was used for search and one missed cleavage as a maximum was permitted. The reverse database search option was allowed, and all peptide data were filtered to satisfy a false discovery rate of 1%. Analysis was performed twice. To further confirm probable protein identities obtained, a search of the peer‐reviewed literature was performed to investigate previous identifications of similar proteins of *Lactobacillus* spp. in other publications.

### Anti‐microbial activity determination of *Lp*‐BS and rhamnolipid

#### Growth inhibition on agar plates by well diffusion assay

Overnight cultures of *Strep. anginosus* group strains and *Ent. faecalis* were adjusted to 1.5 × 10^8^ CFU/ml. Bacterial lawn plates of each strain were prepared by evenly swabbing the prepared bacterial suspension on FAA plates. Wells were then punched into the agar and 200 μl of *Lp*‐BS (50 mg/ml) or rhamnolipid (50, 25, and 12.5 mg/ml in PBS) was dispensed to the wells. Plates were incubated at 37°C and 5% CO_2_ for 24 h. *Esh. coli* NCTC 10,418 and *Staph. aureus* NCTC 8325 (prepared as above but incubated in aerobic conditions) were also examined as reference strains for Gram‐negative and Gram‐positive bacteria, respectively. A clear zone around the inoculation sites was considered as growth inhibition. PBS was added as the vehicle control while vancomycin (1 mg/ml) was used as a positive control against the Gram‐positive strains and gentamicin (1 mg/ml) against the Gram‐negative strain.

#### Minimum inhibitory concentration

The minimum inhibitory concentration (MIC) of the crude *Lp*‐BS and rhamnolipid against the endodontic pathogens (*Strep. anginosus* group strains and *Ent. faecalis* RB17) and reference strains (*Staph. aureus* NCTC 8325 and *Esh. coli* NCTC 10,418) was assessed by broth microdilution susceptibility assay following the method previously described by Gudina et al. [21]. Serial dilutions of *Lp*‐BS in BHI (pH 7) were prepared at 50‐0.01 mg/ml and dispensed to the wells of a 96‐well plate and 10 μl of the respective bacteria (prepared at 1 × 10^8^ CFU/ml and diluted 1:10 in BHI resulting in 1 × 10^7^ CFU/ml) was added to each well. Absorbance at 600 nm was recorded at time zero (*T*_0_) and following incubation at 37°C in 5% CO_2_ for 24 h (*T*_24_). Growth inhibition was calculated using the equation

$$\%\mathrm{Grow}\mathrm{th}\;\;\mathrm{Inhi}\mathrm{biti}{\mathrm{on}}_{c}=\left[1-\left(\mathrm{Ac}/A_{O}\right)\right]\times 100$$

where *Ac* represents the absorbance of the well with a biosurfactant sample concentration *c*, and *A_O_* the absorbance of the growth control well (without biosurfactant); >90% inhibition was determined as the MIC [27].

### Evaluation of *Lp*‐BS and rhamnolipid anti‐adhesive activity by image analysis

Two hundred microlitres of *Lp*‐BS (0, 20, 50 mg/ml in PBS) was added to respective wells of Flacon 8‐well soda‐lime glass chamber slides (Corning), which were wrapped in paraffin and incubated for 24 h at 37°C. To confirm the adherence of *Lp*‐BS, wells were washed twice with PBS and then stained with 20 μl/well fluorescent SYPRO Ruby Protein Gel Stain (Thermo Fisher) for 1 min. The plastic chamber was separated from the glass slide according to the manufacturer's instructions prior to visualisation using an Olympus Provis AX 70 fluorescence microscope with a 20× objective lens. Coating glass slides with 20 mg/ml *Lp*‐BS produced a consistent continuous covering compared to 50 mg/ml *Lp*‐BS, where stained material massed towards the sides of the wells (data not shown). Twenty milligrams per millilitre *Lp*‐BS concentration was, therefore, selected for subsequent evaluation of *Lp*‐BS anti‐adhesive effect. In a separate experiment, bacterial suspensions of *Strep. anginosus* group and *Ent. faecalis* were prepared from overnight culture in PBS (absorbance_600_ = 0.6 in PBS) and 200 μl was pipetted into the wells of glass chamber slides precoated with 10 or 20 mg/ml *Lp*‐BS. Two hundred microlitres of PBS were then added to control wells, and the plates were incubated at 4°C for 2 h. Bacterial suspension was pipetted out and the wells were washed once with 200 μl of PBS.

Preliminary experiments showed that rhamnolipid failed to provide a uniform coating on glass but did so on acrylic discs, thus its anti‐adhesive effects were examined using the latter. Self‐cure acrylic polymer (Bracon) was mixed with self‐cure acrylic monomer (Bracon) at a 2:1 ratio, poured into plastic mould and set at room temperature for 18 h. Discs were soaked in sterile water for 1 week to leach excess monomer and sterilised by autoclaving. Acrylic discs were incubated with either 0.048, 0.097, or 50 mg/ml rhamnolipid or PBS (negative control) at 37°C for 24 h, then washed twice with PBS and transferred into individual wells of a 24‐well plate. One millilitre of each bacterial suspension of *Strep. anginosus* group or *Ent. faecalis* (absorbance_600_ = 0.6 in PBS) was pipetted to each well and incubated at 4°C for 2 h. Non‐adherent bacteria were removed by washing with 1 ml of PBS.

To quantify bacterial attachment to glass or acrylic surfaces, bacteria were fixed with 4% (v/v) formalin for 24 h and then stained with propidium iodide stain (BacLight Bacterial Viability Kit; Life Technologies). Bacteria were visualised using a Leica SP5, AOBS spectral confocal microscope, (Leica; excitation 470 nm, emission 490–700 nm) with a 40× objective lens. Images (.LIF) were collected from five randomly selected areas on each well or acrylic disc and converted to OME‐TIFF files using the COMSTAT/BIOSTAT macro software, for image analysis in ImageJ generating an image for the red (dead) channels. Surface area covered by the fluorescent bacteria was measured and calculated.

### Evaluation of *Lp*‐BS and rhamnolipid anti‐adhesive activity with viable cell count

Glass chamber slides (Corning) coated with either 10 or 20 mg/ml of *Lp*‐BS or acrylic discs coated with 50, 0.097, and 0.048 mg/ml rhamnolipid were prepared and then incubated with bacterial suspension (absorbance_600_ = 0.6 in PBS) for 2 h at 4°C. Bacterial suspension was removed and the wells/acrylic discs washed with 200 μl of PBS. Glass‐attached bacterial cells were harvested using a cell scraper into 1 ml of sterile PBS and vortexed. Discs were placed in sterile plastic bijoux bottles containing 5 ml of PBS and vortexed at 2500 rev/min for 30 s to detach bacteria. Ten‐fold serial dilutions (up to 10^−4^) were prepared in sterile PBS and 50 μl spirally plated onto FAA plates (spiral plater; Don Whitley Scientific), which were then incubated in 5% CO_2_ at 37°C for 24–48 h until colonies were visible to count. Results were expressed as log_10_ CFU per ml from three independent experiments.

### Statistical analysis

Results are presented as mean values ± SD. In comparing anti‐adhesive values of 20 mg/ml *Lp*‐BS coated and untreated control surfaces, statistical differences were evaluated using unpaired *t*‐tests (GraphPad InStat 3 (v3.06). Dunnett's one‐way anova (GraphPad InStat 3, v3.06) was employed to evaluate the statistical difference in comparing anti‐adhesive activity of 50, 0.097, or 0.048‐mg/ml rhamnolipid coated and uncoated surfaces (separate evaluations were performed for each bacterial species examined and all comparisons were made using an uncoated acrylic disc as the control). A *p*‐value of <0.05 was considered significant.

## RESULTS

### Biosurfactant production and characterisation of surface activity

Samples of filtered *L. plantarum* culture broth collected at the different growth curve‐time points showed minimal reduction in surface tension (0–2 mN/m) suggesting the minimal release of biosurfactant into the culture broth (data not shown). Surface tension measurements for the cell‐bound PBS extract indicated surface tension reductions for the 8.5 h mid (17.3 ± 0.5 mN/m, mean ± SD) and 16 h early (11.8 ± 1 mN/m, mean ± SD) exponential phase, and the late stationary phase at 24 h (16.6 ± 0.6 mN/m, mean ± SD) and 48 h (9.3 ± 0.6 mN/m, mean ± SD) when compared to the PBS control. Samples collected at the mid‐exponential phase demonstrated the maximum effect for lowering the surface tension. Based on these results, the mid‐exponential phase sample of *Lp*‐BS was taken forward for further study.

**FIGURE 1:**
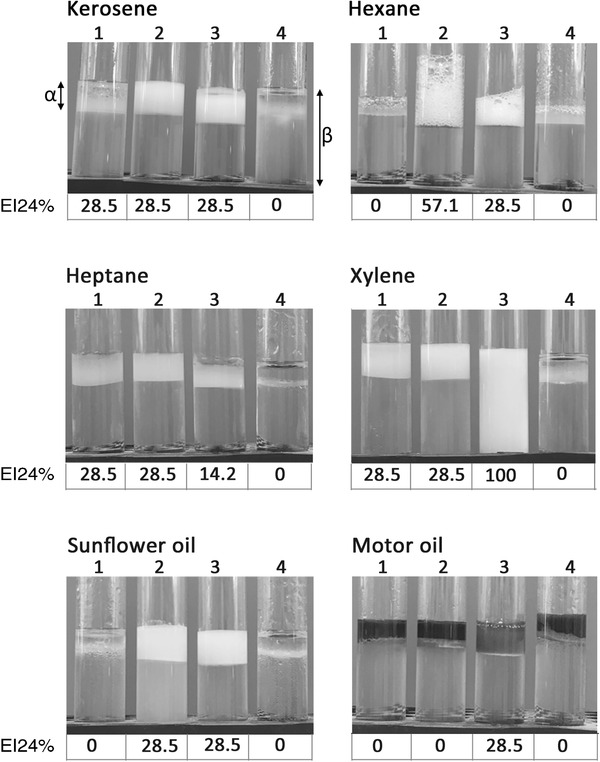
Determination of emulsifying index of a biosurfactant from *Lactobacillus plantarum* (*Lp*‐BS) and rhamnolipid. EI24% was measured for *Lp*‐BS (tube 1) and rhamnolipid (tube 2) against six hydrocarbons. Tween 80 (tube 3) represented a synthetic surfactant control and distilled water (tube 4) was used as the negative control. The symbols α and β indicate the height of the top emulsified layer and the total height, respectively, where EI24% = α/β × 100. Measurements were made in triplicate on three separate occasions and representative images are shown.

Emulsification activity of *Lp*‐BS was compared with rhamnolipid, which is a well‐characterised biosurfactant. Both demonstrated similar ability to emulsify xylene, heptane, and kerosene (EI24 = 28.5%), and this ability was higher compared to the synthetic control Tween 80 (EI24 = 14.5%) (Figure 1). *Lp*‐BS did not emulsify hexane, sunflower oil and motor oil, whereas rhamnolipid demonstrated a powerful emulsifying potential with hexane (EI24 = 57.14%).

### Partial biochemical characterisation of *Lp*‐BS

**FIGURE 2:**
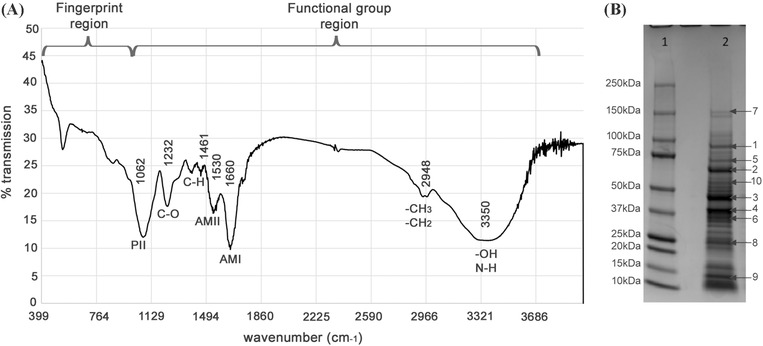
Biochemical characterisation of a biosurfactant from *Lactobacillus plantarum* (*Lp*‐BS). (A) Spectrum produced following Fourier transform infrared spectroscopy (FT‐IR), identifying detectable peaks for functional groups associated with protein (groups labelled with AM) and carbohydrates (groups labelled with P) moieties. (B) Protein separation profile of *Lp*‐BS following SDS‐PAGE and staining with colloidal Coomassie blue. Arrows indicate the bands excised from the gel and processed for liquid chromatography–mass spectroscopy. Lane 1, molecular weight standards; lane 2, *Lp*‐BS

No lipid moiety was identifiable in *Lp*‐BS by thin layer chromatography, which was detectable in rhamnolipid samples, known to contain lipid. For the other colorimetric assays performed, 1 mg of *Lp*‐BS contained 0.186 ± 0.07 mg of carbohydrate and 538 ± 160 μg of protein. The spectra obtained following analysis of *Lp*‐BS by FT‐IR spectroscopy indicated the presence of a protein moiety in *Lp*‐BS, confirmed through the identification of functional groups presenting as strong peaks at 3350 cm^−1^ indicative of OH and NH groups, at 1660.5 cm^−1^ corresponding to C = O stretching (AMI protein band), and at 1530 cm^−1^ typical of N‐H bending (AMII protein band) (Figure 2A). Additionally, small peaks were observed at 2948 cm^−1^ indicative of C‐H stretching and at 1461 cm^−1^ corresponding to C‐H bending (scissor), suggesting the presence of aliphatic chain bonds. A large well‐defined peak at 1062 cm^−1^ was seen correlating with PII (polysaccharides) banding, typical of bond vibrations in C–O–C groups. A small well‐defined peak was detected at 1232 cm^−1^, which represents C–O stretching in sugars.

Following separation of *Lp*‐BS on SDS‐PAGE gel and staining with Coomassie blue, several protein bands were observed with molecular weight extending from 10–150 kDa (Figure 2B). Bands with the highest intensity were cut and analysed by liquid chromatography–mass spectroscopy. Consistent results were obtained from the two repeats. This identified three potential *L. plantarum* proteins involved in the bacterial adhesion (Table 1): elongation factor Tu, enolase elongation factor Tu OS = *L. plantarum* (strain ATCC BAA biosynthesis (Uniprot), previously identified as a mucin adhesion factor of *L. plantarum* CS23 and *L. plantarum* CS24·2 [28]; glyceraldehyde 3‐phosphate dehydrogenase (GAPDH) OS = *L. plantarum* (strain ATCC BAA‐793/NCIMB 8826/WCFS1) OX = 220,668 GN = gapB PE = 3 SV = 1 − [F9UM10_LACPL] (Swiss‐Prot accession number F9UM10), previously reported as a mucin‐binding protein [29]; and Enolase 1 OS = *L. plantarum* (strain ATCC BAA793/NCIMB 8826/WCFS1) OX = 220,668 GN = eno1 PE = 3 SV = 1 − [ENO1_LACPL] of an accession number Q88YH3 (Swiss‐Prot).

**TABLE 1:** Adhesin‐like proteins identified in a biosurfactant from *Lactobacillus plantarum* (*Lp*‐BS) via mass spectrometry

**Protein**	**Uniprot accession no**.	**Band no. in** Figure 2B	**Score in each band respectively**	**% Coverage in each band respectively**	**Function** ^a^	**Reference**
Elongation factor Tu	Q88VE0	2, 3, 4, 5	104.6, 894.0, 136.5, 158.7	64.5, 74.4, 64.3, 64.5	Mucin adhesion factor	[28]
Enolase 1	Q88YH3	3	252.6	69.2	Fibronectin and collagen‐binding protein	[30, 31, 32]
Glyceraldehyde 3‐phosphate dehydrogenase (GAPDH)	F9UM10	3, 4, 6, 8	110.8, 776.2, 311.7, 106.0	63.8, 80.2, 69.7, 63.5	Mucin‐binding protein	[29]

% Coverage: The percentage of the protein sequence covered by identified peptides. Score: The sum of the ion scores of all peptides that were identified.

^a^ Adhesin‐like function is stated here only, other metabolic and catalytic functions are not included.

### Anti‐microbial activity of *Lp*‐BS and rhamnolipid

**FIGURE 3:**
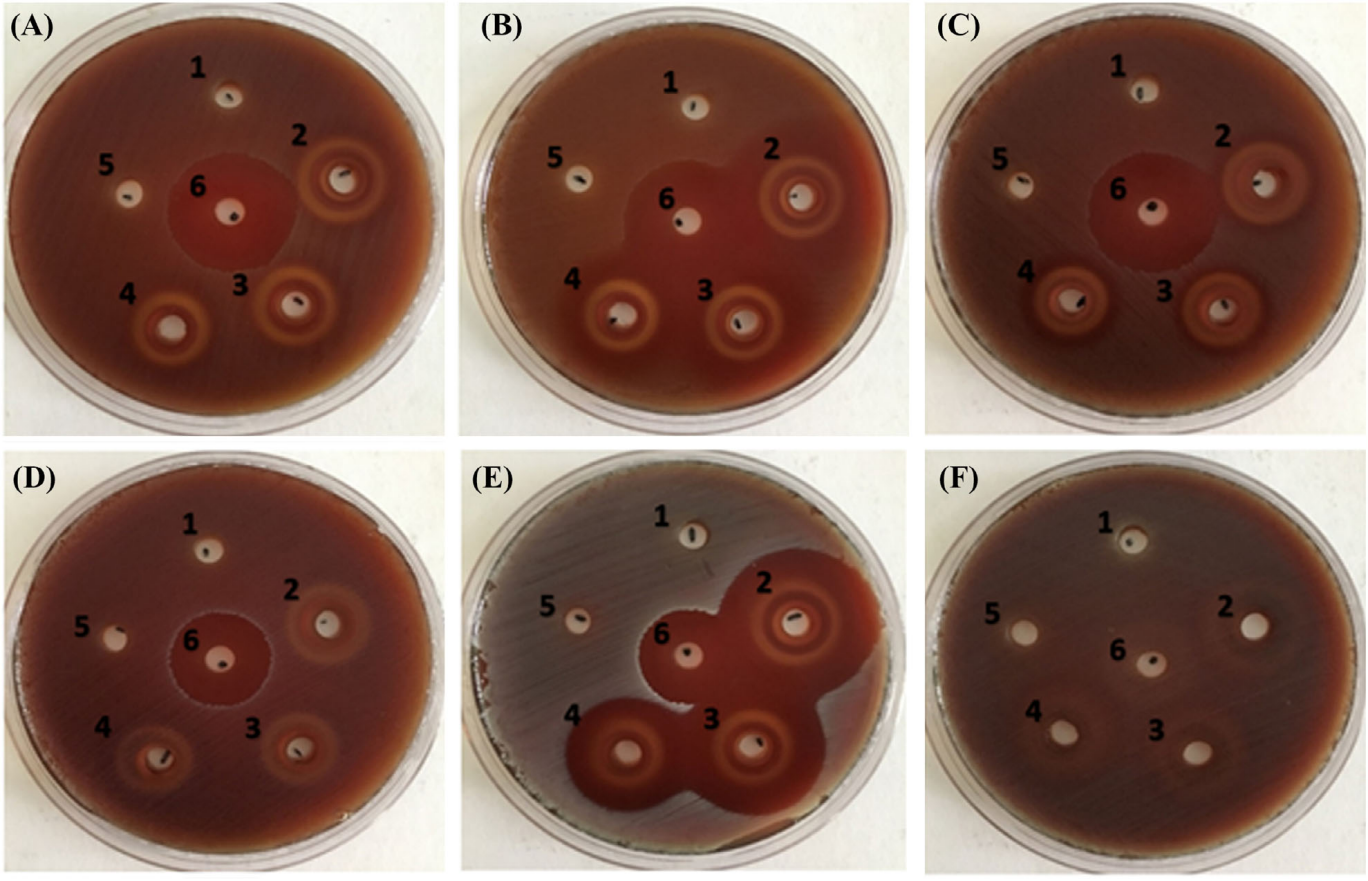
Anti‐microbial activity of a biosurfactant from *Lactobacillus plantarum* (*Lp*‐BS) and rhamnolipid using agar well diffusion assay. No zone of inhibition was monitored for *Lp*‐BS (well 1) against any of the tested pathogens. A clear zone of inhibition was noticed for rhamnolipid against *Strep. anginosus* (A), *Strep. constellatus* (B), *Strep. intermedius* (C), *Staph. aureus* (E), but not *Ent. faecalis* (D) and *Esh. coli* (F). Phosphate buffer (well 5) was used as the vehicle control. Wells 2, 3, and 4 represent rhamnolipid 50, 25, and 12.5 mg/ml, respectively. Well 6 contained vancomycin (1 mg/ml) positive control except for plate F where it contained gentamicin (1 mg/ml). Images are representative of three independent repeats.

Agar diffusion assays indicated that rhamnolipid (50, 25, and 12.5 mg/ml) delivered an anti‐microbial activity against all *Strep. anginosus* group strains and *Staph. aureus*, but no anti‐microbial effect against *Ent. faecalis* or *Esh. coli* was observed (Figure 3). No anti‐microbial activity was detected for *Lp*‐BS at concentrations up to 50 mg/ml. MICs for rhamnolipid were determined for all bacteria examined in this study (Table 2). MICs of 0.048 mg/ml were reported against *Strep. constellatus* and *Strep. intermedius*. *Strep. anginosus* and *Staph. aureus* growth was inhibited completely at a rhamnolipid concentration of 0.097 mg/ml. *Ent. faecalis* required a rhamnolipid concentration of 50 mg/ml to achieve complete inhibition, whilst a concentration of 0.097 mg/ml was able to inhibit *Ent. faecalis* growth by >60%.

**TABLE 2:** Minimum inhibitory concentrations for a biosurfactant from *Lactobacillus plantarum* (*Lp*‐BS) and rhamnolipid against *Strep. anginosus* group members, *Staph. aureus*, *Ent. faecalis*, and *Esh. coli*

	**MIC (mg/ml)**	
**Bacterial strain**	**Rhamnolipid**	***Lp*‐BS**
*Strep. anginosus* 670/95	0.097 ± 0.08	ND
*Strep. constellatus* S08‐07	0.048 ± 0.03	ND
*Strep. intermedius* HW13	0.048 ± 0.04	ND
*Ent. faecalis* RB17	50.00 ± 0.00	ND
*Staph. aureus* NCTC 8325	0.097 ± 0.03	ND
*Esh. coli* NCTC 10418	>50.00 ± 0.00	ND

Data expressed as mean ± SD.

Abbreviations: MIC, minimum inhibitory concentration; ND, not detected.

### Anti‐adhesive effect of *Lp*‐BS precoating of glass slides

**FIGURE 4:**
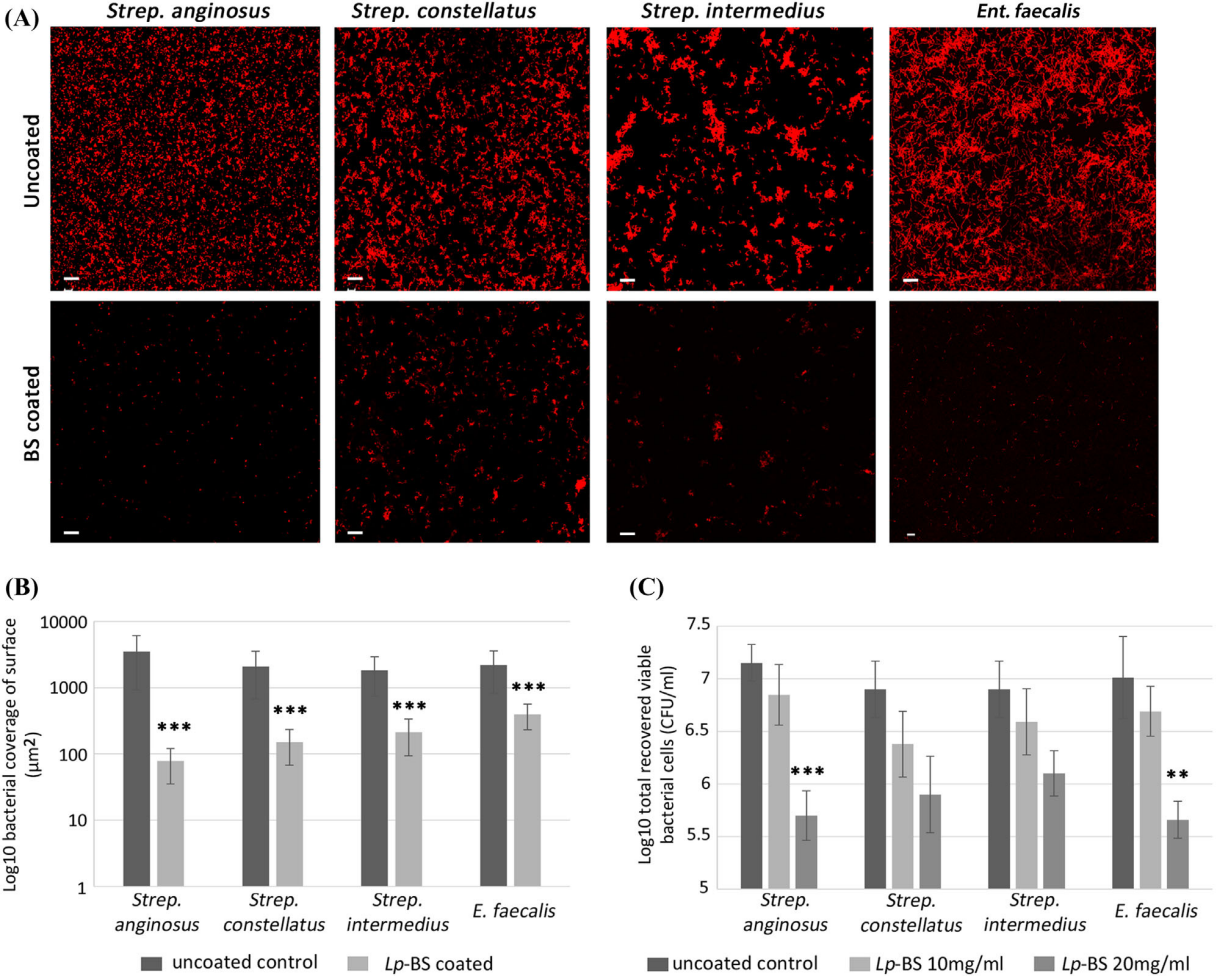
Assessment of the anti‐adhesive activity of a biosurfactant from *Lactobacillus plantarum* (*Lp*‐BS) against *Strep. anginosus* group and *Ent. faecalis* attachment. (A) *Lp*‐BS‐coated and uncoated surfaces of an 8‐well chamber glass slide visualised by confocal laser scanning microscopy after fixation and staining with propidium iodide dye using a 40× objectives. (B) Graph of the statistical analysis of the bacterial‐covered surface area. (C) Bacterial cell recovery of *Strep. anginosus* group and *Ent. faecalis*. Eight‐well chamber glass slides were coated with 10 and 20 mg/ml of *Lp*‐BS, and bacterial attachment was evaluated by recovery of viable cells and enumeration. ***p < 0.001; **p < 0.01; using unpaired *t*‐test. Scale bars represent 200 μm. Error bars represent standard deviation.

Coating the glass slide with 20‐mg/ml suspension of *Lp*‐BS caused a significant (*p* < 0.001) decrease in adhesion of *Ent. faecalis* and the three isolates of the *Strep. anginosus* group when compared to the uncoated control chambers as measured by the log_10_ bacterial covered surface (μm^2^)/field (Figure 4). Further assessment of the anti‐adhesive activity of the crude *Lp*‐BS (10 and 20 mg/ml) was performed by culturing and enumeration of the recovered bacterial cells from the coated and uncoated glass chambers (Figure 4C). The attachment of *Strep. anginosus* and *Ent. faecalis* was significantly reduced (*p* = 0.001 and 0.0049, respectively) by *Lp*‐BS coating (20 mg/ml) when compared to the untreated control. Likewise, reductions for attachment to the coated surface of *Strep. intermedius* (significant, *p* = 0.04) and of *Strep. constellatus* (though insignificant, *p* = 0.05) were observed when compared to the control surface. No significant reduction in adherence of the four bacterial strains to surfaces coated with 10 mg/ml of *Lp*‐BS was observed, indicating a concentration‐dependent anti‐adhesive effect of *Lp*‐BS.

### Anti‐adhesive effect of rhamnolipid

**FIGURE 5:**
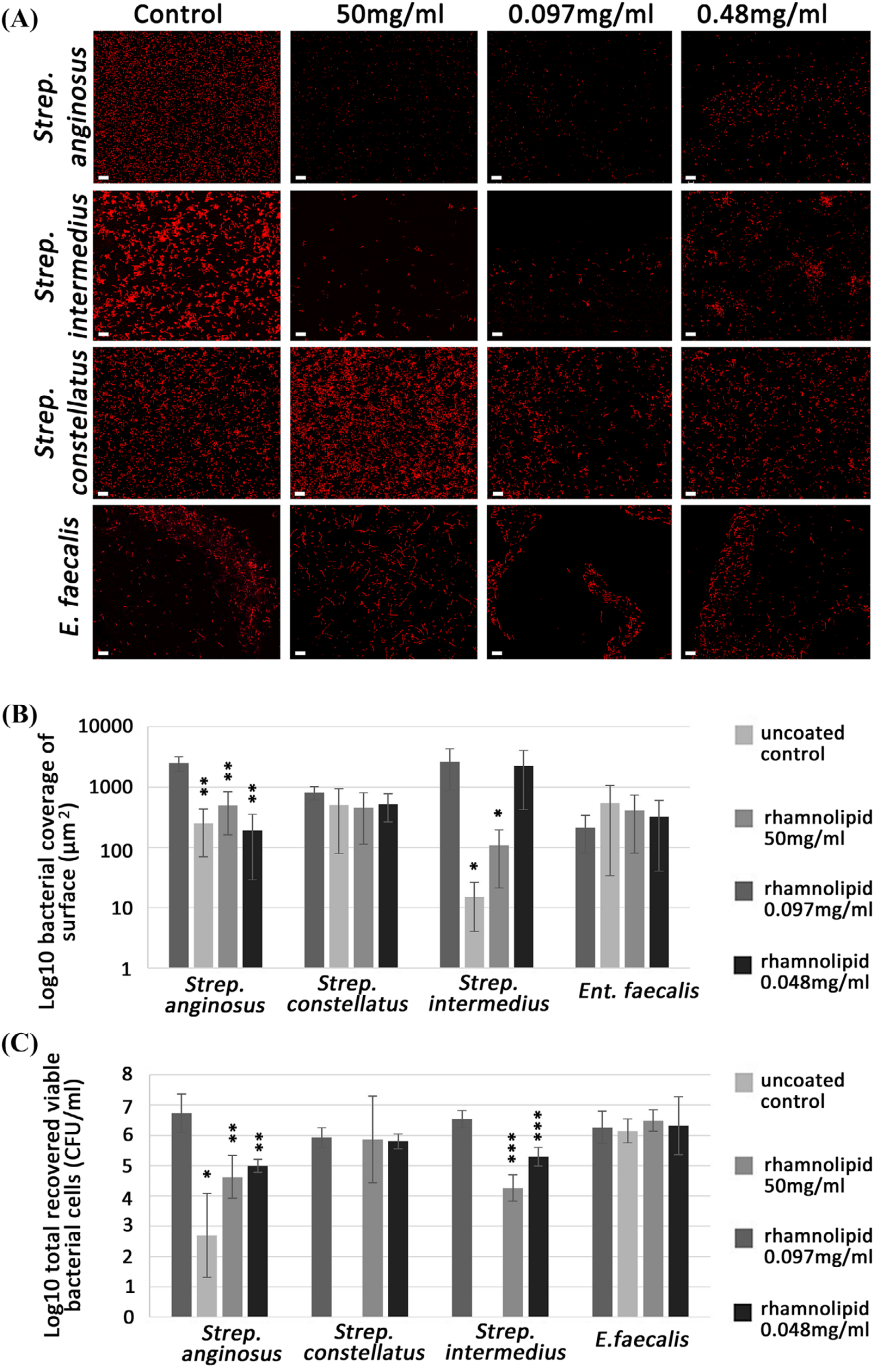
Attachment of *Strep. anginosus* group and *Ent. faecalis* to rhamnolipid‐coated acrylic discs (50, 0.097, and 0.048 mg/ml) and uncoated acrylic discs. (A) Visualisations by confocal laser scanning microscopy. (B) Graph of statistical analysis of the bacterial‐covered surface area. (C) Bacterial cell recovery and enumeration of *Strep. anginosus* group and *Ent. faecalis* from rhamnolipid‐coated and uncoated acrylic coupons. Scale bars represent 200 μm. *p < 0.05; **p < 0.01; ***p < 0.001 using Dunnett's one‐way anova comparing each of the treated group to the untreated control. Error bars represent standard deviation.

A significant reduction in *Strep. anginosus* attachment was observed on surfaces treated with 50, 0.097, or 0.048 mg/ml rhamnolipid solution when compared to untreated control discs (*p* < 0.004, 0.0049, and 0.005, respectively) (Figure 5B). *Strep. intermedius* also showed a significant reduction in adhesion to discs coated with 50 and 0.097 mg/ml rhamnolipid (*p* = 0.040 and 0.044, respectively), but demonstrated no significant decline (*p* = 0.9) in attachment to a disc coated with the lower concentration of 0.048 mg/ml; conversely *Ent. faecalis* and *Strep. constellatus* did not show any statistically significant differences in attachment to the coated and the uncoated control discs (*p* > 0.05) at any concentrations examined (Figure 5B).

Assessment of the anti‐adhesive effects following the recovery of viable cells is shown in Figure 5C. For discs coated with rhamnolipid (50, 0.097, and 0.048 mg/ml), a statistically significant reduction was observed in the recovery of viable *Strep. anginosus* cells (*p* = 0.02, 0.0022, and 0.0049, respectively) when compared to the untreated control. No viable *Strep. intermedius* cells were recovered from 50‐mg/ml coated discs. Coating discs with 0.097 and 0.048 mg/ml rhamnolipid resulted in a significant reduction (*p* < 0.0001) in the number of viable *Strep. intermedius* cells. No statistically significant reduction in viable *Strep. constellatus* was observed when recovered from discs coated with rhamnolipid 0.097 and 0.048 mg/ml (*p* = 0.995 and 0.97, respectively), but viability reduced to zero at the high concentration of 50 mg/ml. No statistically significant differences were recorded in *Ent. faecalis* recovered from discs coated with rhamnolipid at 50, 0.097, and 0.048 mg/ml (*p* = 0.9576, 0.7806, and 0.9947, respectively).

## DISCUSSION

In this study, we successfully extracted and conducted preliminary characterisation of a high molecular weight cell‐bound biosurfactant from the probiotic *L. plantarum* NCIMB8826 strain. Biosurfactant production kinetics have been studied for various bacteria [6, 33] where their production is associated with (a) growth, (b) production in response to growth‐restrictive conditions, (c) resting cells production, and (d) growth‐supplemented production [33]. *Lp*‐BS production was greatest during the mid‐exponential growth phase, suggesting that production is related to bacteria in the growth phase. The observed progressive reduction in biosurfactant production across the stationary phase may be due to the consumption and limitation of important nutritional elements or because *L. plantarum* biomass had reached a critical density that negatively influenced biosurfactant production [34]. The ability to produce biosurfactant in a nutrient‐limited environment has relevance for the use of probiotics within the nutrient‐limited endodontic canals.

*Lp*‐BS was initially characterised through measurements of its surface activity. Our results compare favourably with measurements for cell‐associated biosurfactants characterised by several *Lactobacillus* spp. [6, 24, 35] that have been reported to have an ability to lower surface tension within the range of 12–33 mN/m. *Lp*‐BS also demonstrated an emulsification index comparable to that of *L. plantarum* CFR2194 biosurfactant [24]. Knowledge is limited regarding the chemical composition of this cell‐bound biosurfactant. However, the identification of the glycoprotein nature of *Lp*‐BS shows similar chemical composition with other studies on *L. plantarum* CFR2194 [24], *L. pentosus* CECT4023 [36], *Lactococcus lactis* 53 [6], *L. agilis* CCUG31450 [25], *L. acidophiles* [37], and *L. acidophilus* ATCC 4356 [22].

Anti‐microbial analysis showed a lack of anti‐microbial activity of *Lp*‐BS against selected endodontic pathogens and reference Gram‐positive and Gram‐negative bacteria, in contrast to that observed with rhamnolipid. In accordance with the current finding, no anti‐microbial effect was reported for the cell‐bound *L. plantarum* biosurfactant against *Staph. aureus*, even at the relatively high concentrations of 100 mg/ml [38]. In contrast, biosurfactants extracted from two *L. plantarum* strains successfully demonstrated anti‐microbial activity against several food‐borne microbes [39]. Although anti‐microbial activity of the glycolipid rhamnolipid has been widely investigated and reported [40, 41], there have been no reports of its anti‐microbial effect against the endodontic pathogens *Strep. anginosus* group and *Ent. faecalis*. While *Strep. anginosus* group members responded to low rhamnolipid concentrations (MIC = 0.097 mg/ml), *Ent. faecalis* was found to be more resistant and required a high concentration of 50 mg/ml to be inhibited completely. A wide range of rhamnolipid MICs has been reported against different strains of *Ent. faecalis*. For instance, no anti‐microbial effect against clinical and reference strains of *Ent. faecalis* was noticed for rhamnolipid extracted from *P. aeruginosa* MR01 at 512 μg/ml whilst rhamnolipid that was produced from *P. aeruginosa* MASH1 demonstrated significant inhibitory effect against the two strains of enterococcus at a lower MIC value of 64 μg/ml [39]. By contrast, rhamnolipid extracted from *P. fluorescence* showed the ability to suppress around 50% of *Ent. faecalis* growth at 50 μg/ml [430]. A lower inhibitory concentration (MIC = 4 μg/ml) against *Ent. faecalis* was reported for *P. aeruginosa* LBI rhamnolipid [40]. The mechanisms by which biosurfactants exert their anti‐microbial effect have not been fully elucidated. However, studies have proposed mechanisms comparable to those of conventional antibiotics [44]. For instance, the ability to fit into and disintegrate the pathogen cell membrane has been demonstrated and they have been shown to induce cell death via protein synthesis interference [45, 46].

Bacterial tissue colonization and infection are mediated via interaction between host tissue extracellular matrix proteins, such as collagen, fibronectin, mucin, laminin, and fibrinogen, with specific bacterial surface molecules [30, 47, 48]. Probiotics have been proposed to function in a similar manner where their mode of action may be via adherence with specific host tissue surface receptors, leading to competitive exclusion of pathogenic bacteria binding to the same attachment sites and thereby blocking the first step of bacterial infection [32, 49]. Indeed, adhesion proteins have been identified in several *Lactobacillus* spp. and have been considered as an essential element of the probiotic anti‐microbial effects [50]. A surface displaced α‐enolase of *L. plantarum* LM3 has been shown to bind to both fibronectin [30] and collagen I [32]. Likewise, it has been proposed that GAPDH of *L. plantarum* LA 318 plays a role in probiotic adhesion to the mucin of the human colon [29]. Such mucin‐binding activity has also been reported for elongation factor Tu from *L. plantarum* CS23 and *L. plantarum* CS24.2 which subsequently blocks the attachment of *Esh. coli* and *Salmonella* species. A similar scenario can be envisaged for their derived biosurfactants. Anti‐adhesiveness is another valuable feature of certain biosurfactants, where they exert their effect by adsorption to the substratum surfaces or infection sites resulting in a change in the surface hydrophobicity and/or interference with the course of microbial adhesion [6]. *Lactobacillus* strains have been shown to produce proteinaceous biosurfactants of the surfactin‐type having a marked potential to hinder pathogen adherence to surfaces in addition to their catalytic and metabolic functions (hence labelled as ‘moonlighting’ proteins) [20, 21, 31]. This can potentially influence bacterial adherence to root canal surfaces and tubules and subsequently might minimise the chance of treatment failure. To the best of our knowledge, no previous studies have investigated the effect of *L. plantarum* NCIMB 8826 biosurfactant as an anti‐microbial/anti‐adhesive agent against endodontic pathogens and *Ent. faecalis*.

A promising approach of coating surfaces with biosurfactants has been reported as an effective anti‐infective strategy of particular relevance to biofilm infections and anti‐biotic resistance [17, 38]. *Lactobacillus* spp. biosurfactants that are rich in unspecified proteins have been reported to exert powerful affinity in binding to a range of surfaces [20]. The current identification of the three surface adhesins in *Lp*‐BS mixture supports the potential usefulness of *Lp*‐BS as anti‐adhesive biomolecules of therapeutic significance. *Lp*‐BS successfully impeded *Strep. anginosus* group and *Ent. faecalis* attachment to a glass surface in a concentration‐dependent manner. In accordance with the current finding, the glycoprotein biosurfactant of *L. plantarum* CFR 2194 has been demonstrated to efficiently inhibit the adhesion of food‐borne pathogens (*Esh. coli* ATCC 31705, E*sh. coli* MTCC 108, *Salmonella typhi*, *Yersinia enterocolitica* MTCC 859 and *Staph. aureus* F 722) [24]. In parallel with the present study finding, *Staph. aureus* CMCC 26003 attachment was inhibited by 50% following coating the surface with 50 mg/ml of *L. plantarum* 27172 cell‐bound biosurfactant with no anti‐microbial activity reported at the mentioned concentration [38].

The mechanism of how biosurfactants work as anti‐adhesive agents is still unclear. It has been hypothesised that these agents can change the surface properties, such as hydrophobicity, making it more resistance to bacterial attachment [51]. In addition, they may also act by manipulating gene expression of certain bacterial genes. For instance, a *Lactobacillus* cell‐bound biosurfactant has been found to hinder the ability of *S. mutans* strains, a major cariogenic pathogen, to form biofilm via down‐regulation of dental adhesion genes glucosyltransferases and fructosyltransferase (gtfB, gtfC, and ftf) [52]. A 29‐kDa protein was purified from a biosurfactant mixture isolated from *L. fermentum* RC‐14 that showed significant anti‐adhesive effect against the virulent uropathogenic *Ent. faecalis* 1131. The purified protein demonstrated 100% identity to a collagen‐binding protein, which was isolated from *L. reuteri* NCIB 11951 in a previous work [53]. Subsequently, the same research group identified collagen‐binding proteins from biosurfactant mixtures isolated from *L. fermentum* RC‐14, *L. rhamnosus* GR‐1, and *L. casei* [54]. The involvement of the three identified *Lp*‐BS ECM‐binding proteins (elongation factor Tu, Enolase 1, and GAPDH) [28–32] may give the rationale of the observed anti‐adhesive potential of *Lp*‐BS. However, deeper investigations are required to establish the connection between these proteins and the *Lp*‐BS anti‐adhesive effect. The different coating ability of the two biosurfactants identified in the present study may be attributed to the differences in chemical composition and hydrophobicity between rhamnolipid and *Lp*‐BS, and consequently may explain why rhamnolipid failed to form a coating on glass surfaces. However, when coated to acrylic surfaces, the attachment of *Strep. intermedius* and *Strep. anginosus* was hindered in the presence of rhamnolipid in a concentration‐dependent manner. In one study, rhamnolipid of *Strep. mitis* was found able to hamper the adherence of *Strep. mutans* to a glass surface. Most notably, this anti‐adhesive effect is increased in the presence of fluid shear forces similar to those expected to occur in the oral cavity [55]. Rhamnolipids have been suggested to exert anti‐biofilm activity via changing cell surface polarity [56]. The lack of an anti‐adhesive potential of rhamnolipid for *Ent. faecalis* observed in this study might be due to a stronger adhesion tendency of the bacteria to the acrylic surface or the ability of *Ent. faecalis* to use rhamnolipid as a conditioning layer for initial attachment. Similarly, it was demonstrated that rhamnolipid did not affect the adhesion of *P. aeruginosa* E26 strain to borosilicate coupons [57]. The chemical diversity of microbial biosurfactants may explain the heterogeneity of their biological effects [33]. For instance, the glycoprotein cell‐bound biosurfactant extracted from *L. plantarum* CFR 2194 was shown to exert significant anti‐microbial activity [24]. The functional groups of its FT‐IR spectrum are identical to that of *Lp*‐BS; however, they differ in their fingerprint region demonstrating a different chemical structure of the two glycoproteins. Similarly, the cell‐bound glycoprotein biosurfactant of *L. agilis* demonstrated significant anti‐microbial activity [25] but it shares a different FT‐IR fingerprint region to that of *Lp*‐BS, which lacks anti‐microbial effect. This finding supports the strain‐specific effect of the probiotics and indicates that each strain may need to be tested individually for anti‐microbial activity [58]. Similarly, the chemical heterogeneity of rhamnolipid and the strain‐specific effect may play a role in the variation of its anti‐microbial effect [40]. In addition, the variability in the lipid composition of the microbial cell membrane, which has been shown to be the target of rhamnolipid, may influence its anti‐microbial potential [59, 60].

In summary, the anti‐adhesive activity of *Lp*‐BS, which is plausibly related to the identified three adhesin‐like proteins, together with the anti‐microbial effect of rhamnolipid, opens promising possibilities for establishing the foundation of novel therapies in the prevention of infection during endodontic therapy. This could include incorporating rhamnolipid and/or *Lp*‐BS into a ‘smart’ hydrogel base to ensure the controlled release and a mode of application into the pulp or the root canals. Such formulation may serve as a supporting mechanical matrix, which is required for the pulp capping agents, aiding pulpal cells’ adherence and multiplication and hence permiting normal pulp tissue development. They can also be applied as co‐irrigant solutions to provide prophylactic surface coating against microbial colonisation of the root canal. Further biophysiochemical optimisation of the proposed formulations is required. Additional strategies are welcomed in the face of increasing antibiotic resistance. However, it is acknowledged that further investigations need to be performed to assess the toxicity/immunogenicity of these bacterial extracts on human tissues.

## AUTHOR CONTRIBUTIONS

**Conceptualization**: Zahraa A. Hashim, Jean‐Yves Maillard, Melanie J. Wilson, and Rachel J. Waddington. **Methodology**: Zahraa A. Hashim, Jean‐Yves Maillard, Melanie J. Wilson, and Rachel J. Waddington. **Validation**: Zahraa A. Hashim. **Formal analysis**: Zahraa A. Hashim. **Investigation**: Zahraa A. Hashim, Jean‐Yves Maillard, Melanie J. Wilson, and Rachel J. Waddington. **Resources**: Zahraa A. Hashim, Jean‐Yves Maillard, Melanie J. Wilson, and Rachel J. Waddington. **Data curation**: Zahraa A. Hashim. **Writing**: Zahraa A. Hashim and Rachel J. Waddington. **Writing—review and editing**: Zahraa A. Hashim, Jean‐Yves Maillard, Melanie J. Wilson, and Rachel J. Waddington. **Supervision**: Rachel J. Waddington, Jean‐Yves Maillard, and Melanie J. Wilson. **Project administration**: Rachel J. Waddington. **Funding acquisition**: Zahraa A. Hashim.

## CONFLICT OF INTEREST

The authors confirm that there are no conflicts of interest or relationship, financial or otherwise, associated with this study.
